# Paclitaxel synergizes with exposure time adjusted CD22-targeting immunotoxins against B-cell malignancies

**DOI:** 10.18632/oncotarget.16141

**Published:** 2017-03-11

**Authors:** Fabian Müller, Stephanie Stookey, Tyler Cunningham, Ira Pastan

**Affiliations:** ^1^ Laboratory of Molecular Biology, Center for Cancer Research, National Cancer Institute, National Institutes of Health, Bethesda, MD, USA; ^2^ Department of Hematology and Oncology, University Hospital Erlangen, Erlangen, Germany; ^3^ MD Program, University of North Caroline, Chapel Hill, NC, USA; ^4^ MD/PhD Program, University of Miami, Miller School of Medicine, Miami, FL, USA

**Keywords:** CD22-targeted immunotoxin, mantle cell lymphoma, targeted therapy, combination therapy, paclitaxel

## Abstract

CD22-targeted recombinant immunotoxins (rIT) are active in hairy cell leukemia or acute lymphoblastic leukemia (ALL), but not in mantle cell lymphoma (MCL) patients. The goal was to enhance rIT efficacy *in vivo* and to define a strong combination treatment. Activity of Moxetumomab pasudotox (Moxe) and LR combined with paclitaxel was tested against MCL cell lines *in vitro* and as bolus doses or continuous infusion in xenograft models. In the KOPN-8 ALL xenograft, Moxe or paclitaxel alone was active, but all mice died from leukemia; when combined, 60% of the mice achieved a sustained complete remission. Against MCL cells *in vitro*, LR was more active than Moxe and the cells had to be exposed to rIT for more than 24 hours for them to die. To maintain high blood levels *in vivo*, LR was administered continuously by 7-day pumps achieving a well-tolerated steady plasma concentration of 45 ng/ml. In JeKo-1 xenografts, continuously administered LR was 14-fold more active than bolus doses and the combination with paclitaxel additionally improved responses by 135-fold. Maintaining high rIT-plasma levels greatly improves responses in the JeKo-1 model and paclitaxel substantially enhances bolus and continuously infused rIT, supporting a clinical evaluation against B-cell malignancies.

## INTRODUCTION

We have developed recombinant immunotoxins (rIT) which consist of an antibody fragment fused to a truncated *Pseudomonas* exotoxin A [1]. The first CD22-targeting rIT BL22 (CAT-3888) is active against B-cell non-Hodgkin lymphoma (B-NHL) cell lines *in vitro* [2] and achieves an objective response rate of 81% in patients with hairy cell leukemia (HCL) [3]. However, BL22 fails to produce objective responses in pediatric B-cell acute lymphoblastic leukemia (B-ALL) [4] or mantle cell lymphoma (MCL) [3]. The affinity matured BL22-variant HA22 or Moxetumomab pasudotox (Moxe) [5] is 10-fold more potent than BL22 *in vitro* and achieves an overall response rate of 86% in HCL patients [6] and of 33% in pediatric B-ALL [7]. HCL patients treated with Moxe regularly develop neutralizing antibodies which likely reduce the anti-leukemic activity in these patients [6]. To decrease immunogenicity, we generated HA22-PE24 (named LR) [8] which lacks domain II and therefore major B- [9] and T-cell epitopes [10]. In addition, LR is more active than Moxe or BL22 against various ALL and lymphoma cell lines and against patient-derived chronic lymphocytic leukemia (CLL) [8]. LR has not yet been developed clinically.

MCL is a rare subtype of B-NHL of 6% [11]. New treatment options include the Burton's Tyrosine kinase (BTK)-inhibitor Ibrutinib [12, 13], the proteasome inhibitor Bortezomib [14–16], the immune-modulator Lenalidomide [17–19], and the mTORC-inhibitor Temsirolimus [13, 20] which are active in patients with relapsed/refractory MCL; various combinations of these small molecules with current standard treatment regimens are in clinical testing [11, 21–23]. Despite recent progress in treatment options and the introduction of intensified chemotherapy regimens with autologous stem cell transplantation leading to a doubling of progression-free survival of MCL patients over the past two decades, most patients still relapse and MCL remains an incurable disease [22].

We recently showed that the activity of CD22-targeting rIT against ALL cells *in vitro* and *in vivo* improved substantially when the cells were exposed longer to CD22-targeting rIT [24]. Because rITs have a short plasma half-life in mice [8] and men [7], blood levels fall quickly after a bolus dose. The rIT exposure time of ALL cells growing in murine bone marrow (BM) achieved by bolus doses was too short for them to die; when high rIT blood levels were maintained by repeated injections, *in vivo* efficacy was greatly improved [24].

Mesothelin-targeting rITs are enhanced by various small molecules [25–27] or the chemotherapeutic drug paclitaxel [28–30]. Paclitaxel enhances the efficacy of bolus doses of mesothelin-targeted rIT *in vivo* [28, 29, 31], which was translated in a new clinical trial testing the combination (NCT02810418). Whether *in vivo* efficacy of CD22-targeting rIT is enhanced by paclitaxel has not been determined previously.

Here, we tested whether the addition of paclitaxel would improve treatment outcome in the KOPN-8 xenograft model [24]. To extend our studies toward MCL, various cell lines were tested *in vitro* and LR was found to be more active than Moxe. The time that MCL cells needed to be exposed to either rIT for them to die varied largely from hours to days. To enable continuous drug delivery *in vivo* by 7-day osmotic pump, the rIT-formulation buffer was optimized to ensure protein stability, which then allowed for the comparison of bolus dose and continuous administration and finally the combination with paclitaxel in a newly established systemic JeKo-1 xenograft model.

## RESULTS

### Paclitaxel/rIT-combination achieves durable remissions in an ALL xenograft model

**Figure 1:**
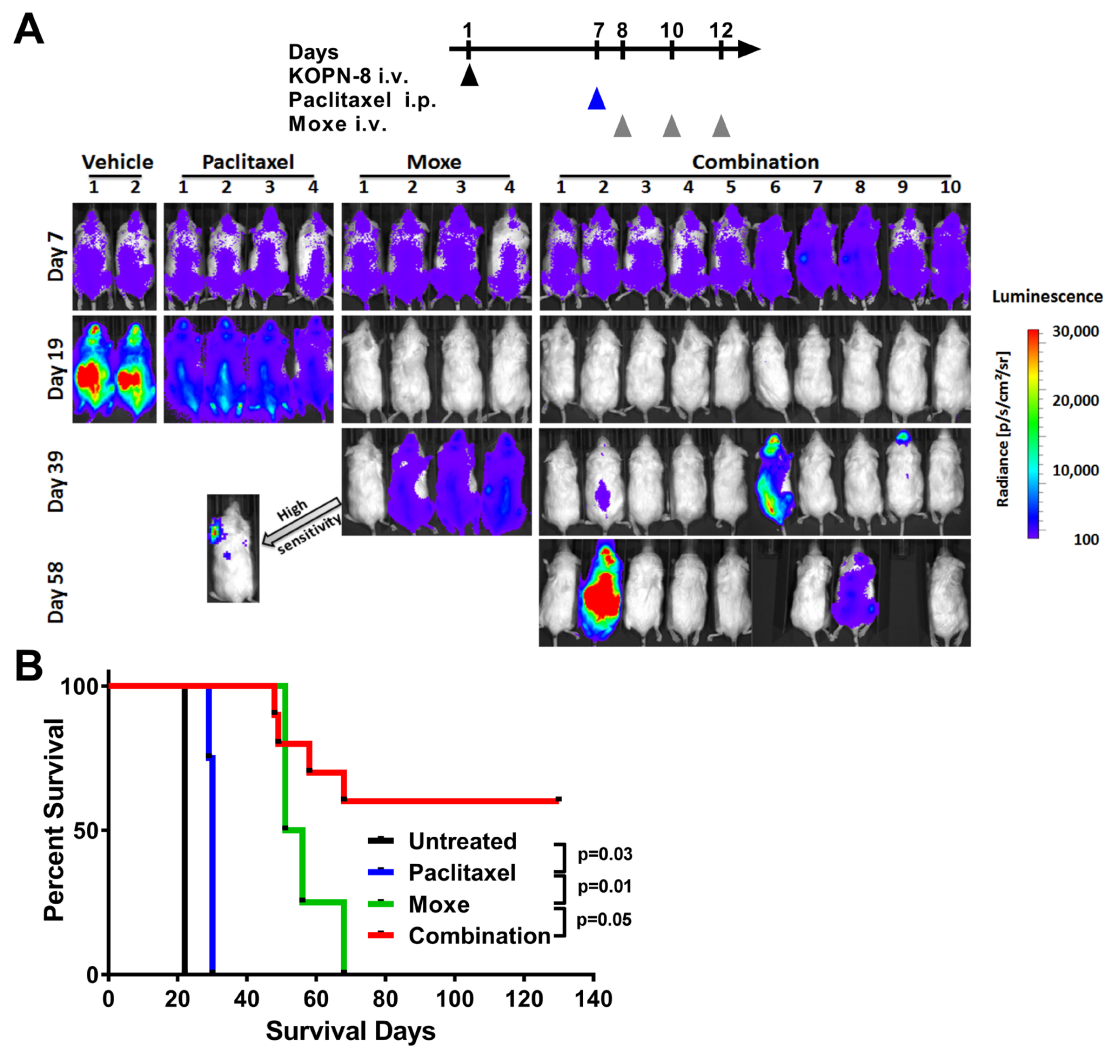
Paclitaxel enhances ***in vivo*** efficacy of CD22-targeting immunotoxin in KOPN-8 xenografts. NSG mice bearing luciferase positive KOPN-8 cells were treated with a single dose of 25 mg/kg paclitaxel i.p. on day 7, 3 doses of 0.4 mg/kg Moxe QOD from day 8, or the combination. **A**. Bioluminescence signal was measured at the indicated days. All images were taken with identical camera settings (binning “medium”, exposure time 1 second). Signal intensity is shown as radiance from 100 (blue) to 30,000 (red). Mice without any detectable bioluminescence were imaged a second time using “high sensitivity” camera settings (binning “high”, exposure time 1 minute). The arrow points towards the “high sensitivity” image of mouse one of the Moxe group. **B**. Animal survival is shown as a Kaplan Meyer curve, the significance was determined by log-rank test.

To search for combination therapies which enhance the *in vivo* efficacy of CD22-targeting rITs, we used our recently developed KOPN-8 xenograft model [24]. After i.v. injection, the KOPN-8 cells grow in the murine BM and three bolus doses of Moxe every other day (QOD) reduce the KOPN-8 BM infiltration below the detection limit of flow cytometry. However, all mice relapse and die from leukemia [24]. To track treatment responses in real time, KOPN-8 cells were transduced with luciferase and green fluorescent protein (GFP) and single cloned. After i.v. injection, the disease burden was measured by determining the bioluminescence. In this KOPN-8 model, the combination of paclitaxel and Moxe improved responses greatly (Figure 1A). Before start of treatment on day 7, all mice showed a similar bioluminescence signal. In accordance with the known KOPN-8 BM infiltration [24], the bioluminescence was found in close proximity to bones. Untreated mice progressed rapidly as shown by a steep increase in bioluminescence. For mice receiving a single dose of 25 mg/kg paclitaxel i.p. on day 7 bioluminescence increased between days 7 and 19, but was lower than the bioluminescence of control mice on day 19. Three i.v. bolus doses of 0.4 mg/kg Moxe QOD given from day 8 resulted in an abrogation of the bioluminescence signal on day 19 but the bioluminescence recurred in all these mice indicating expected leukemia relapse. The mice which had received the combination of Moxe and paclitaxel showed an abrogation of the bioluminescence on day 19. In mice 2, 6, and 9 on day 39 and in mouse 8 on day 58 bioluminescence recurred, indicating leukemia relapse which was confirmed by flow cytometry analysis of the BM post mortem. The remaining six mice stayed bioluminescence free until day 130 when the experiment was terminated and no evidence was found for residual KOPN-8 cells in the murine BM by flow cytometry (data not shown).

The time the KOPN-8 bearing mice survived after treatment correlated directly with the course of their bioluminescence signal (Figure 1B). Untreated mice survived on average until day 22, mice receiving paclitaxel alone until day 30, and mice receiving Moxe alone until day 53. The six mice lacking any bioluminescence after receiving the combination lived until the experiment was terminated on day 130. We conclude that the combination of paclitaxel and Moxe improves responses in the KOPN-8 xenograft model.

### LR is more active than Moxe *in vitro*

To test the rIT and paclitaxel combination in an MCL xenograft model, we first compared Moxe and LR for their *in vitro* activity against MCL cell lines by WST-8 assays. Table 1 shows that LR was more active than Moxe against all MCL cell lines with IC_50_s ranging from 0.3 ng/ml to 6 ng/ml (6 pmol/l to 120 pmol/l). The LR-activity was higher by 1.7-fold against Rec-1, 2.4-fold against JeKo-1, 2.4-fold against JVM-2, 3.0-fold against Mino, and 3.3-fold against Z-138 (Table 1). The data show that the deletion of all but the furin cleavage site of domain II results in an increased activity of CD22-targeting rIT against MCL cell lines which is in line with the increased activity of LR against patient derived CLL [8].

**Table 1:** Activity of Moxe and LR against MCL cell lines

	Moxe-IC50 [ng/ml]	LR-IC50 [ng/ml]	Fold-Difference*	*p*-value
Rec-1	0.3+/−0.05	0.2+/−0.03	1.7	0.08
Jeko-1	1.2+/−0.1	0.5+/−0.04	2.4	0.002
JVM-2	1.6+/−0.2	0.7+/0.13	2.4	0.01
Mino	2.6+/−0.8	0.9+/−0.15	3.0	0.11
Z-138	6.0+/−1.8	1.9+/−0.5	3.3	0.10

IC_50_ was determined by WST8 assay after exposure to serial dilutions of HA22 or LRGGS for 3 days. *Fold-difference of activity was corrected for molecular weight difference between Moxe and LR. Data represents means of three independent experiments, error is shown as SEM, p-values were determined by paired t-tests.

### MCL cell lines need highly variable rIT-exposure time for them to die

**Figure 2:**
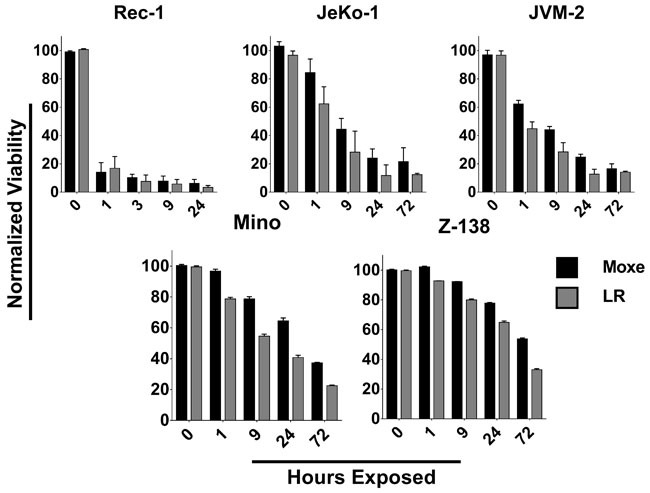
The time for rITs to reach maximal cytotoxic activity is highly variable Indicated MCL cell lines were exposed to 140 ng/ml Moxe (black) or equimolar LR (grey) for various times, washed, replated, and cell-viability determined after a total of 3 days. Viability of untreated cells was set to 100%, 0% was defined as real 0. Each bar represents the average percent living cells of three independent experiments, error is shown as SEM.

Whether the activity of rIT against MCL cells depends on the time the cells are exposed to rIT was tested by treating MCL cell lines with 140 ng/ml Moxe or equimolar LR for various times, cells were washed and replated in complete medium without rIT and viability determined three days after assay initiation, enough time for the cells to die if they were exposed to a lethal rIT dose. Figure 2 shows that the exposure time needed to kill more than 50% of the cells varied among the cell lines from less than one hour to three days. Killing of more than 50% of the Rec-1 cells was reached after less than 1 hour, of JeKo-1 or JVM-2 cells after 9 hours, of Mino cells after 24 hours and of Z-138 cells after 72 hours. In Rec-1 and JVM-2 there was no significant difference between Moxe and LR, while JeKo-1 (*p* = 0.01), Mino (*p* = 0.02), and Z-138 (*p* = 0.03) were killed faster by LR than by Moxe. The longer the MCL cells are exposed to rIT *in vitro* the more likely they die. This exposure time varies from hours to days.

### Continuous administration improves responses 10-fold

**Figure 3:**
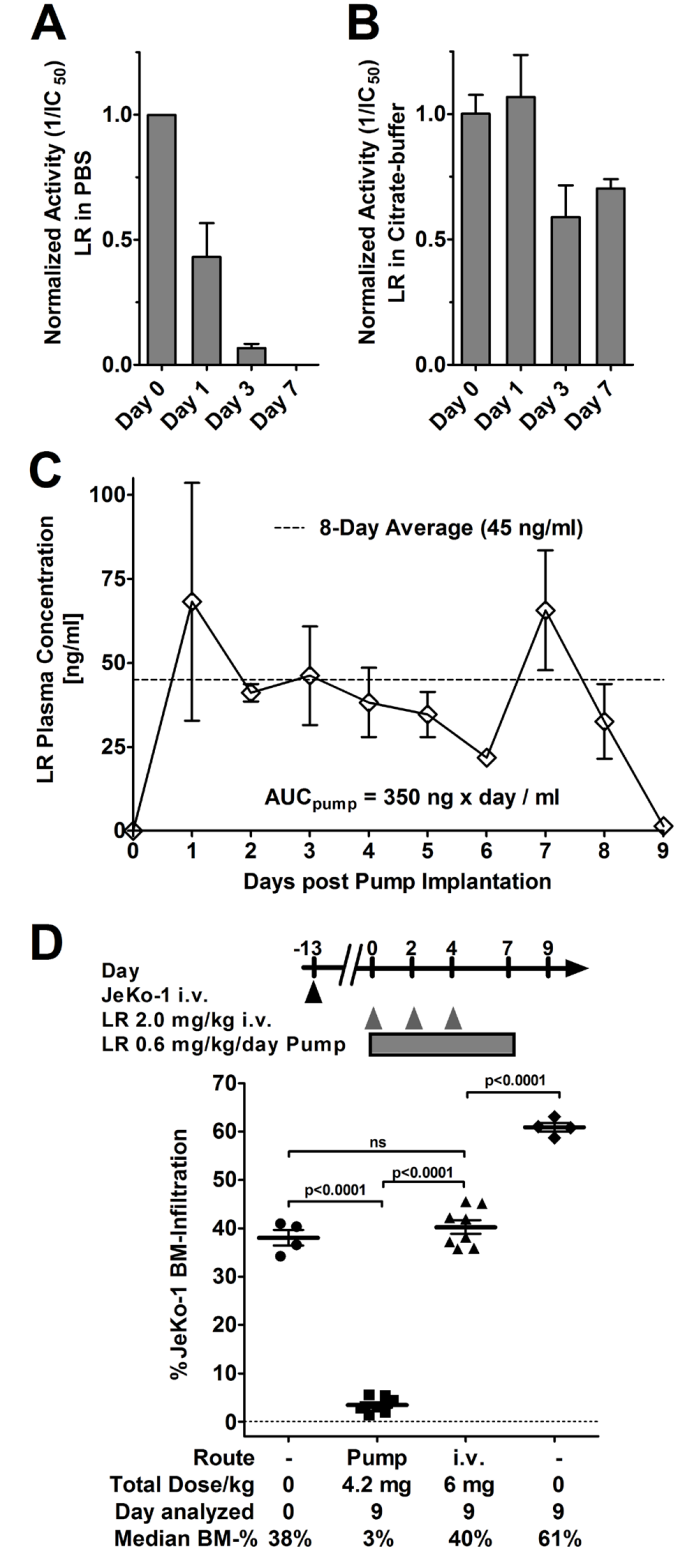
LR in citrate buffer is stable, pump-administration achieves high serum levels increasing activity by 10-fold LR at 2 mg/ml in standard buffer PBS **A**. or citrate buffer **B**. were incubated at 37°C for the indicated times, frozen at -80°C, and activity of each sample determined by WST8 assays. Activity of an immediately frozen aliquot (day 0) was set to 1 to which the remainder were normalized. Bars indicate average normalized IC_50_, errors are shown as SEM. **C**. Mice were implanted with an ALZET osmotic pump containing LR at 1 mg/ml in citrate buffer on day 0. From day 1, blood was collected, the plasma separated by centrifugation, and the LR concentration was determined by WST8 assays. Each symbol represents the average LR plasma concentration of 3 mice, except day 6 (only 2 mice), error is shown as SEM (except day 6), AUC as determined by Graph Pad Prism, v.6.01. The average plasma concentration is indicated as a dashed line. **D**. Some mice bearing systemic Jeko-1 were euthanized on day 0 and the JeKo-1 BM infiltration determined. The remainder were either implanted with a 7-day ALZET osmotic pump containing 1 mg/ml LR in citrate buffer and a pump rate of 0.5 μl/h, treated with 3 doses of 2.0 mg/kg LR QOD i.v. from day 0, or treated with vehicle. The treated mice were sacrificed on day 9 and their BM was analyzed for JeKo-1 cells by flow cytometry. Symbols indicate individual mice, lines represent mean JeKo-1 BM-infiltration, error as SEM. Significance determined by unpaired t-test as ns (not significant) or as indicated.

Because rITs have a short serum half-life of approximately 15 minutes in mice [8], but JeKo-1 cells need more than 9 hours of rIT exposure for 50% of the cells to die, we hypothesized that *in vivo* efficacy may improve by maintaining high rIT blood levels. In mice, continuous administration can be achieved by implanting an osmotic pump into the peritoneal cavity where it remains for seven days at body temperature. We tested LR stability in the standard rIT-formulation PBS at 37°C. The data in Figure 3A show that LR in PBS loses activity rapidly. LR-activity fell 2.5-fold below initial activity within one day, fell 9-fold within three days, and was not detectable after seven days. We empirically tested several buffer formulations for their capability to stabilize LR. Stabilization was most efficient in the clinically used Moxe formulation (32 mM citrate, 0.65% Tween80, 5 mM EDTA) where LR was stable for up to seven days (Figure 3B). LR-activity on day 1 was similar to the activity at assay initiation and was less than 2-fold reduced by days 3 or 7, respectively. The buffer exchange from PBS to citrate-buffer itself did not change LR-activity (Supplementary Figure S1). We conclude that LR in the citrate buffer is sufficiently stable to treat mice with a 7-day osmotic pump.

The steady state plasma concentration reached in mice by pump-administrated LR was then determined from plasma serial dilutions using WST-8 assays. Over an 8-day period post-implantation, LR-plasma concentration was on average 45 ng/ml (Figure 3C) correlating with an area under the curve (AUC) of 350 ng x day/ml_Plasma_. This steady state plasma concentration was higher than the IC_50_ of any of the MCL cell lines tested.

A systemic JeKo-1 xenograft model was used to test whether continuously administered LR was more active than bolus doses. Within 14 days after i.v. injection, JeKo-1 cells grow predominantly in the BM and proliferate at later stages also in other organs including the spleen and the liver [32]. The data in Figure 3D show that continuous infusion substantially increased efficacy of LR compared to bolus dose administration. At treatment start defined as day 0, the rate of JeKo-1 BM-infiltration as determined by flow cytometry was on average 38% and increased to 61% by day 9 in untreated mice. Three i.v. bolus doses of 2 mg/kg LR on days 0, 2, and 4 resulted in an infiltration rate of 40% on day 9, which was significantly lower than in untreated mice (*p* < 0.0001), but not different from the rate of JeKo-1 BM-infiltration at treatment start. Continuously infused LR reduced the JeKo-1 BM-infiltration by 13-fold to an infiltration rate of 3% which was significantly lower than the infiltration after three bolus doses (*p* < 0.0001). Maintaining continuously high LR blood levels improved responses greatly.

### Enhancement by paclitaxel is minor *in vitro* but substantial *in vivo*

**Figure 4:**
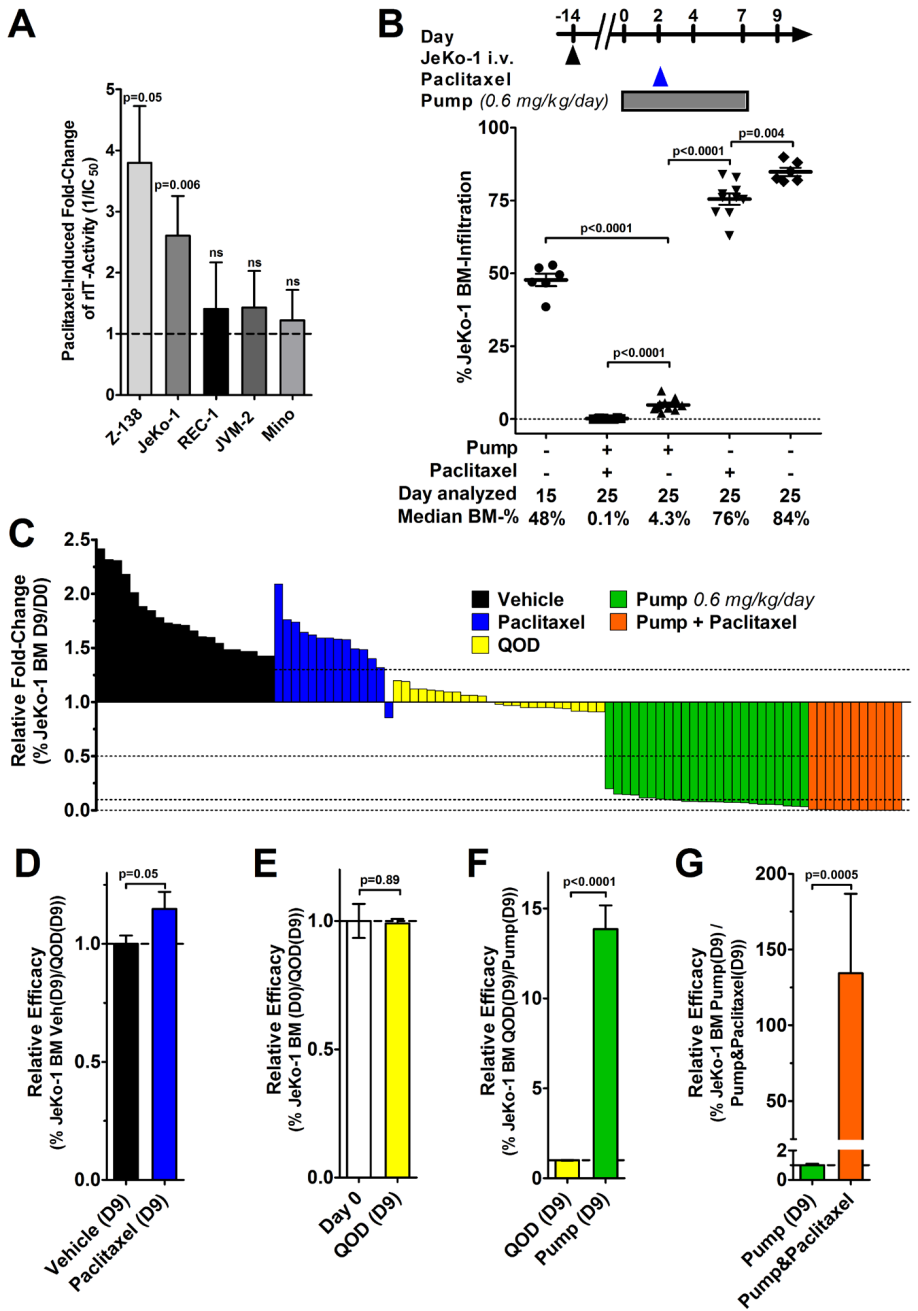
Minor ***in vitro*** enhancement contrasts with a substantial improvement of ***in vivo*** efficacy by the addition of paclitaxel. **A**. MCL cell lines were treated with LR and various concentrations of paclitaxel; after 72 hours, cells were stained with Annexin V-PE and 7-AAD and viability was determined by flow cytometry. The bars represent the mean IC_50_-fold changes when paclitaxel was added. Fold-change values were only included if paclitaxel alone reduced cell viability by at least 15% (indicating that paclitaxel at this concentration was active) but not more than 85% (non-linear regression becomes unreliable below this threshold). The fold-changes were summarized from at least three independent experiments; errors as SEM, p-values were determined by unpaired t-tests. **B**. Some JeKo-1 bearing mice were analyzed for BM infiltration at treatment start on day 0 and the remainder treated with either vehicle, a single dose of 25 mg/kg paclitaxel i.p. on day 2, implanted with a 7-day ALZET osmotic pump containing 1 mg/ml LR in citrate buffer from day 0, or the combination of pump and paclitaxel. Symbols indicate individual mice, lines represent mean JeKo-1 BM-infiltration, errors as SEM, p-values were determined by unpaired t-test. **C**. The graph summarizes 95 mice from a total of 6 individual experiments. Each bar represents one mouse and its fold-change of JeKo-1 BM infiltration 9 days after the indicated treatment relative to the average BM-infiltration at the treatment start of the corresponding experiment control on day 0. The color indicates treatment type as vehicle (black), Taxol mono (blue), 2.0 mg/kg LR i.v. QOD (yellow), LR 0.6 mg/kg/day by 7-day pump (green), and the combination of LR by pump and paclitaxel (red). **D**.-**G**. To determine the relative efficacy, the average BM-infiltration in the indicated groups was set to 1 and the relative fold-change was determined as (D) vehicle treated on day 9 (D9) vs. paclitaxel (D9), as (E) day 0 vs. QOD (D9), (F) as QOD (D9) vs. pump-administrated LR (D9), and as (G) pump-administrated LR without vs. with paclitaxel (D9). Color codes in (D-G) are as indicated in (C), the bars show the mean relative fold-change, errors are shown as SEM, p-values were determined by unpaired t-tests.

Before testing the combination of pump-administered LR and paclitaxel *in vivo*, we determined whether paclitaxel enhances LR *in vitro* by WST8 assays. Four of five MCL cell lines were sensitive to paclitaxel alone at concentrations achieved in murine organ tissue after a single dose of 25 mg/kg (Supplementary Figure S2 [33]). The sensitive cell lines showed IC_50_s of 2.0 nM for JeKo-1, 2.4 nM for Z-138, 4.1 nM for Mino, and 5.8 nM for JVM-2, whereas the less sensitive Rec-1 showed an IC_50_ of 153 nM. When paclitaxel and LR were combined *in vitro*, activity improved in two of five cell lines (Figure 4A, Supplementary Figure S3). Paclitaxel enhanced LR-activity 3.7-fold against Z-138 and 2.4-fold against Jeko-1, while LR-activity against Rec-1, JVM-2, or Mino remained unchanged.

For *in vivo* testing, JeKo-1-bearing mice were implanted with a 7-day osmotic pump on day 0, 14 days after tumor inoculation. To allow for wound healing after the surgery, paclitaxel was injected i.p. 2 days after the pump implantation. Mice were euthanized on day 9 and their BM analyzed. As shown in Figure 4B, mice at treatment start had on average a JeKo-1 BM-infiltration of 48% which increased to 84% on day 9 in vehicle treated mice. Treatment with paclitaxel alone reduced JeKo-1 BM-infiltration significantly to 76% on day 9 (*p* = 0.004). Compared to treatment initiation, the continuously infused LR reduced the JeKo-1 BM-infiltration by 11-fold (*p* < 0.0001). Combining the continuously infused LR with paclitaxel further decreased the Jeko-1 BM-infiltration to 0.1% (*p* < 0.0001).

To enable a comparison of six independent experiments, we determined the fold-change in JeKo-1 BM infiltration of each of 95 individual mice nine days after treatment initiation relatively to the average JeKo-1 BM infiltration at treatment start within their respective experiment (Figure 4C). The data show that vehicle and Taxol-treated mice progressed, QOD-treated mice showed a stable BM-infiltration, which decreased when LR was administered by pump and decreased even further by the addition of paclitaxel. To directly compare activity of treatment groups, the relative efficacy was mathematically determined (Figures 4D-4G). Treating mice with a single dose of paclitaxel on day 2 was on average 1.15-fold more active than vehicle in reducing BM-infiltration on day 9 (p = 0.05, Figure 4D). Compared to infiltration at treatment start, three bolus doses LR QOD did not change the JeKo-1 BM-infiltration (*p* = 0.89, Figure 4E). Exchanging bolus doses with continuous administration resulted on average in a 14-fold increased efficacy (*p* < 0.0001, Figure 4F). Therefore, a well-tolerated total amount of 84 μg LR given by continuous infusion is substantially more active than the 120 μg LR given as three bolus doses QOD.

By the addition of paclitaxel to continuously administered LR, the responses improved on average by 135-fold over pump administration alone (p = 0.0005, Figure 4G). Of note, in the BM of all mice that had received the combination, viable JeKo-1 cells were still detectable on day 9 by flow cytometry when experiments were terminated. Due to pump properties, the experiments had to be stopped 10 days after implantation and therefore, effects of the treatment on animal survival were not determined. The efficacy of continuously infused LR is substantially enhanced by paclitaxel.

## DISCUSSION

Searching for a molecule that enhances CD22-targeting rIT *in vivo*, we found the combination with paclitaxel to eradicate leukemia cells in 60% of KOPN-8-bearing mice which survive tumor-free for months. With the goal to test the efficacy of this combination in an MCL xenograft, we found *in vitro* (i) that LR was more active than Moxe against MCL cells and that (ii) MCL cells had to be exposed to CD22-targeting rITs for a highly variable time for them to die. Due to a short serum half-life of 15 - 20 minutes, rIT plasma concentrations after an i.v. bolus dose fall within less than 2 hours to inactive levels. In accord with the needed exposure time of 9 hours for 50% of the Jeko-1 cells to die *in vitro*, response to CD22-targeting rIT improves more than 10-fold in the JeKo-1 xenograft model when i.v. bolus doses are exchanged for continuous infusion by osmotic pumps. The addition of paclitaxel to the optimized continuous rIT-administration increases efficacy more than 100-fold over continuous LR alone.

An *in vivo* enhancement of mesothelin-targeting rITs by paclitaxel has been described in xenograft models of solid tumors including the mesothelin-expressing pancreatic cancer cell line KLM-1 [28], breast cancer cell line HCC-70, gastric cancer cell line MKN-28 [29], and the cervical cancer cell line KB [31]. Similar to our findings with CD22-targeting rIT, enhancing effects for paclitaxel have been shown for mesothelin-targeting rITs with domain II [31] and without [28, 29], resulting in a new clinical trial testing the efficacy of the LMB-100 in combination with Nab-paclitaxel for patients with mesothelin expressing solid tumors (NCT02810418). Taken together the data suggest that paclitaxel enhances rITs *in vivo* independent from the target or domain II-status. In the KOPN-8 model, CD22-targeting rIT alone reduces tumor burden below the detection level by bioluminescence or flow cytometry and only a few cells remain, presumably responsible for late relapse and animal death [24]. It is possible that paclitaxel kills these few remaining cells specifically shown here. An alternative explanation which is supported by the JeKo-1 data and the data on mesothelin-targeted rITs is that the two drugs have a highly synergistic mechanism in killing the target cells and the few remaining cells in the KOPN-8 model are sensitized to LR.

We currently do not understand the mechanism behind the exceptional *in vivo* synergy of paclitaxel and rITs. *In vivo* results with the KB tumors suggested that reduced shedding of surface mesothelin by the paclitaxel treatment reduced decoy receptors produced by the tumor, ultimately increasing delivery of rIT [31]. Improvement of drug delivery after paclitaxel treatment was not shown in a KLM-1 model [28], making the reduction of decoy receptors as the reason for improved rIT-efficacy here unlikely. That only a little CD22 is shed from the cell surface [34] also argues against reduced shedding as a major mechanism for the *in vivo* enhancement of CD22-targeted rIT. While the enhancement by paclitaxel *in vivo* is substantial, *in vitro* effects for the enhancement of Mesothelin-targeting rITs have been small or absent [35]. We similarly find a comparably small two to three-fold enhancement of immunotoxin activity against only two of the five MCL cell lines *in vitro*. Immunotoxins arrest protein synthesis, ultimately leading to a fall of the anti-apoptotic protein MCL1, which destabilizes mitochondria and activates the intrinsic apoptosis [36], while paclitaxel activates the extrinsic apoptosis [37]. Both apoptosis pathways converge in late apoptotic events which may explain the *in vitro* synergy. Alterations in the expression levels of the regulators of programmed cell death, the Bcl-2 family proteins are common in hematologic malignancies [38] which may explain the variation in the rIT-enhancing effects of paclitaxel *in vitro* [37]. That the >100-fold enhancement of continuously administered LR in JeKo-1-bearing mice by paclitaxel is distinctively higher than the only two-fold enhancement *in vitro* suggests a distinct mechanism responsible for the exceptional *in vivo* synergy and indicates that *in vitro* tests may not be predictive. The *in vivo* mechanism of the paclitaxel induced rIT-enhancement remains unexplained.

In the first clinical trial, the CD22-targeting rIT BL22 achieved a high rate of objective responses in HCL patients but failed to show activity in CLL, follicular lymphoma, MCL [3], and ALL [4]. Objective responses in ALL patients increased after increasing the affinity to CD22 by 10-fold [7], but were still below the expected response rate [39]. Recent data from ALL xenograft models led to the concept that responses might improve if high rIT blood levels are maintained longer [24]. The previous data suggest that differences in the complex trafficking of Pseudomonas exotoxin A [40] may explain the differences in the time cells need to be exposed to immunotoxin for them to die. We describe here that CD22-targeting rITs show an exposure time dependent activity also against MCL cells *in vitro* and translate these findings into an MCL xenograft model. Because of the rapidly falling rIT blood levels after a bolus dose in patients, the data suggest that responses in B-NHL patients might improve if rIT was given continuously.

In summary, continuous infusion as opposed to bolus doses substantially improves response in an MCL xenograft model which corroborates our previous findings in ALL, emphasizing that a change of the current bolus dose administration to continuous infusion may substantially improve response rates in MCL patients. The exceptionally strong synergy between paclitaxel and CD22-targeting rIT makes this combination a promising candidate for future clinical testing.

## MATERIALs AND METHODS

### Reagents

Moxe [5] and LR [8] were produced as previously described. For the last step in the rIT purification process [42], the size exclusion chromatography, elution buffer PBS, pH 7.4 was changed to a citrate formulation (32 mM citrate pH 6.0, 5 mM EDTA, 0.65% Tween 80). Clinical grade paclitaxel in Cremophore (6 mg/ml) was purchased from Teva Pharmaceuticals (#00703-4764-01). For *in vitro* application, paclitaxel was diluted in RPMI to indicated concentrations and for *in vivo* application it was diluted to 1.25 mg/ml in PBS, pH 7.4.

### Cell lines

The Jeko-1, JVM-2, and REC-1 cells were described previously [43]. The Mino and Z-138 cells were kindly provided by Dr. Louis Staudt, NCI/NIH. The B-cell precursor (BCP-) ALL cell line KOPN-8 [24] was transduced with a luciferase and GFP containing lentivirus, sorted for the top 5% GFP expression twice, and single-cloned. All cells were grown in RPMI supplemented with 10% fetal bovine serum, 100 U penicillin, and 100 mg streptomycin (Invitrogen).

### Cell assays

Cytotoxicity was determined by WST-8 (Dojindo Molecular Technologies, Rockville, MD, #CK04-20) as described [26]. 5,000 cells/well were incubated with various rIT concentrations for 72 hours. WST-8 reagent was added, absorbance was measured 2 hours later, values were normalized between Cycloheximide (10 μg/ml final, Sigma-Aldrich, St. Louis, MO, #C4859-1ML) and untreated control and, non-linear regression to obtain IC_50_ concentrations was done using GraphPad Prism v6.01.

For *in vitro* apoptosis assays by flow cytometry, 1 million cells/ml were incubated with 2.8 nM rIT for various times, cells washed twice after the indicated incubation times, resuspended in complete RPMI, and transferred to a new plate. Seventy-two hours after assay initiation, cells were stained with 7-AAD/Annexin-PE, and measured with a FACS Calibur (BD). Results were analyzed with FlowJo software (Tree Star).

### Stability testing

LR in PBS or in Citrate buffer was concentrated to 1 mg/ml using Amicon Ultracell spin columns (Millipore, #UFC803024). To test for stability at 37°C, tubes were incubated in a heating block for indicated times and aliquots frozen at -80°C until used. The remaining rIT-activity was determined by WST8 assays.

### Animal studies

Animals were handled according to NIH guidelines; studies were approved by the NCI Animal Care and Use Committee.

Ten million JeKo-1 or five million KOPN-8 cells were injected on Day 1 via tail vein into 6- to 8-week-old NSG mice (NOD.Cg-*Prkdc*scid *Il2rg*tm1Wjl/SzJ). One mg/ml LR in citrate buffer was loaded into 7-day ALZET osmotic pumps dispensing at 0.5 μl/h. The pumps were surgically implanted into the peritoneal cavity following manufacturer's instructions. rIT was i.v. injected as three bolus doses of 0.4 mg/kg Moxe QOD or 2.0 mg/kg LR QOD. Paclitaxel was given as a single dose of 25 mg/kg paclitaxel i.p. To assess treatment responses, mice were euthanized 10 days after osmotic pump implantation. BM was extracted by flushing femurs. Mouse-derived tissue was F_c_-receptor blocked with anti-murine CD16/32, JeKo-1 cells were stained with anti-human-CD20-FITC, viability was determined using 7-AAD, and cells were analyzed on a FACS Calibur.

To measure the rIT plasma concentration, mice were implanted with osmotic pumps and two groups of three mice each were bled alternatingly QOD by puncture of the mandibular vein. Thirty μl blood/mouse/puncture was collected in heparinized tubes, plasma separated by centrifugation, and stored at -80 °C until used. Thawed, plasma samples were serial diluted in RPMI and tested for cytotoxic activity against Rec-1 cells by WST-8. The plasma dilution factor at which 50% Rec-1 cells were growth inhibited was determined by non-linear regression and the corresponding LR plasma concentration was extrapolated from a standard curve generated by treating Rec-1 with serial dilutions of LR.

### Luciferase imaging

D-luciferin (150 mg/kg VivoGlo, Promega, # P1043) was injected i.p., mice anesthetized with Isoflurane, and images taken 5 minutes after injection using a Xenogen IVIS-100 (Caliper).

### Statistics

Statistical analyses were performed with Graph Pad Prism v6.01 as paired or unpaired t-tests as indicated and as log-rank tests for animal survival.

## SUPPLEMENTARY MATERIALS FIGURES



## Abbreviations

ALL: Acute lymphoblastic leukemia
AUC: area under the curve
B-ALL: B-cell ALL
BCP-ALL: B-cell precursor ALL
BM: bone marrow
B-NHL: B cell non Hodgkin's lymphoma
BTK: Bruton's Tyrosine kinase
CLL: chronic lymphocytic leukemia
GFP: green fluorescent protein
HCL: hairy cell leukemia
MCL: mantle cell lymphoma
Moxe: Moxetumomab pasudotox
QOD: every other day
rIT: recombinant immunotoxin.
