# Supplementary material for: Paclitaxel synergizes with exposure time adjusted CD22-targeting immunotoxins against B-cell malignancies
Source: Oncotarget. 2017 Mar 11;8(19):30644–55. doi: 10.18632/oncotarget.16141 (PMC5458156; doi:10.18632/oncotarget.16141)
Supplement: Supplementary file 1 [file oncotarget-08-30644-s001.pdf]

# Paclitaxel synergizes with exposure time adjusted CD22-targeting immunotoxins against B-cell malignancies

## Supplementary Material

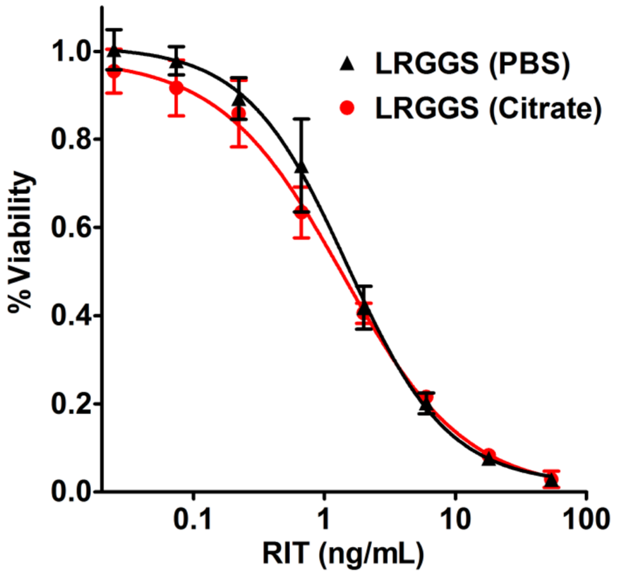


| | LRGGS (PBS) | LRGGS (Citrate) |
|---|---|---|
| IC50 | 1.492 | 1.378 |

**Supplementary Figure 1: Exchange to citrate buffer does not influence LR activity.** LR in PBS was buffer exchanged on PD10 columns. Activity of LR which was buffer exchanged from PBS to citrate buffer (32 mM citrate, 5 mM EDTA, 0.65% Tween 80, pH 6.0) was compared to LR which was exchanged from PBS to PBS by WST8 growth inhibition assays. Shown is one of three experiments with similar results using the cell line Z-138. Symbols indicate means of triplicates, error as SD, non-linear regression to determine IC50 was done with Graph Pad Prism v6.01.

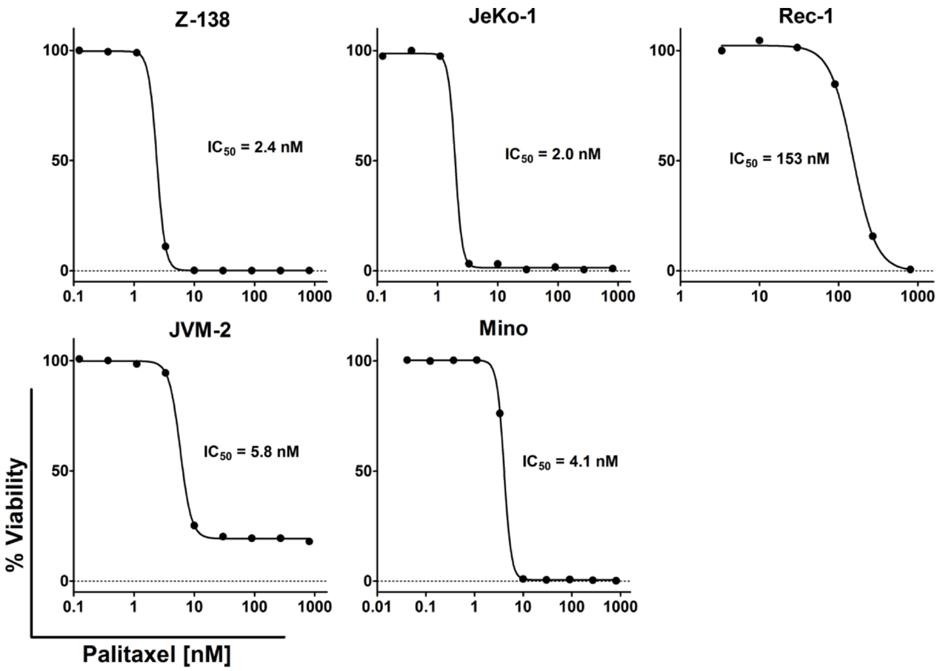


**Supplementary Figure 2: Paclitaxel is active against MCL cell lines *in vitro*.** MCL cell lines were treated with serial dilutions of Paclitaxel. After 72 hours, cells were stained with Annexin V-PE and 7-AAD and viability was determined by flow cytometry. 100% percent living cells within each assay was set to the internal paclitaxel- and rIT-untreated control, to which all other samples were normalized. 0% viability was defined as true 0.

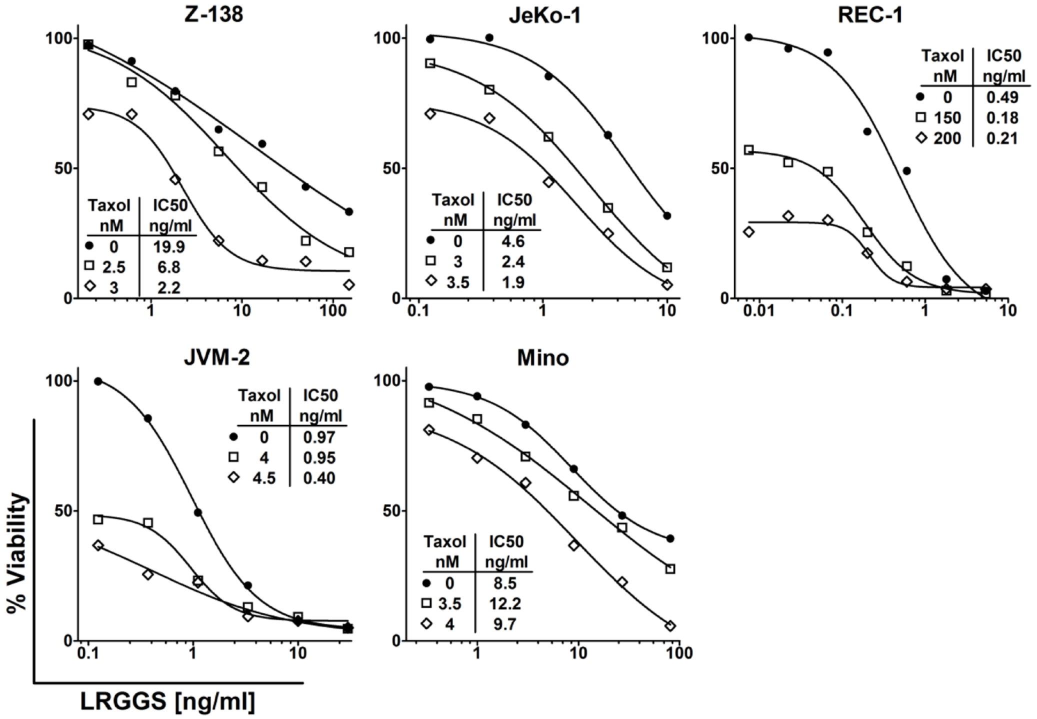


**Supplementary Figure 3: Paclitaxel enhances LR against two of five MCL cell lines *in vitro*.** MCL cell lines were treated with LR in combination with various concentrations of paclitaxel. After 72 hours, cells were stained with Annexin V-PE and 7-AAD and viability was determined by flow cytometry. 100% percent living cells within each assay was set to the internal paclitaxel- and rIT-untreated control, to which all other samples were normalized. 0% viability was defined as true 0. All figures are representatives of at least three independent experiments with similar results.
